# Dietary fat quantity and quality in relation to general and abdominal obesity in women: a cross-sectional study from Ghana

**DOI:** 10.1186/s12944-020-01227-5

**Published:** 2020-04-11

**Authors:** Sufyan Bakuri Suara, Fereydoun Siassi, Mahama Saaka, Abbas Rahimi Foroshani, Sara Asadi, Gity Sotoudeh

**Affiliations:** 1 Department of Community Nutrition, School of Nutritional Sciences and Dietetics, International Campus, Tehran University of Medical Sciences, Number 21, Dameshgh St. Vali-e Asr Ave., Tehran, 1416753955 Iran; 2 Department of Community Nutrition, School of Nutritional Sciences and Dietetics, Tehran University of Medical Sciences, Hojatdost street, Naderi street, Keshavarz Blv, Tehran, Iran; 3 Department of Nutritional Sciences, School of Allied Health Sciences, University for Development Studies, Post Office Box 1350, Tamale, Ghana; 4 Department of Statistics and Epidemiology, School of Public Health, Tehran University of Medical Sciences, Tehran, Iran

**Keywords:** Atherogenicity index, Thrombogenic index, Lipophilic index, Obesity, Overweight

## Abstract

**Background:**

Although relationships between obesity and total fat and fat types have been widely examined, the associations between the relative proportions of fatty acids calculated in the form of indices and obesity/overweight are lacking. The objective of this study was to assess associations between dietary fat quality indices and odds of obesity/overweight in women from Ghana.

**Methods:**

In this cross-sectional study, dietary information was obtained using 24-h dietary recall. The odds of obesity were evaluated across quintiles of specific DFQ indices [atherogenicity index (AI), thrombogenic index (TI), hypo- and hypercholesterolemic fatty acids ratio (h/H), omega-3 to omega-6 polyunsaturated fatty acids ratio (∑ω-3/∑ω-6), polyunsaturated fatty acids/saturated fatty acids ratio (PSR), dietary lipophilic index (LI) and percentage of energy from total fat (TF)].

**Results:**

After adjustment for covariates, general obesity and overweight were inversely associated with ∑ω-3/∑ω-6 ratio (OR: 0.63; 95% CI: 0.24–1.63; P for trend (P) = 0.005) and positively with TI (4.14; 95% CI: 1.78–9.66; *P* = 0.01) and LI (2.49; 95% CI: 1.14–5.43; P = 0.01). The odds of abdominal obesity based on waist circumference (WC) were significantly higher among participants in the fifth quintile (Q) compared with those in the first Q of AI (1.24; 95% CI: 0.56–2.74; P = 0.01), TI (4.14; 95% CI: 1.78–9.66; *P* = 0.009), LI (2.11; 95% CI: 0.98–4.55; *P* = 0.02) and TF (1.59; 95% CI: (0.73–3.46; *P* = 0.003). Similarly, waist to height ratio (WHtR) was positively associated with AI (2.89; 95% CI: 1.32–6.31; *P* = 0.04), TI (2.65; 95% CI: 1.22–5.76; *P* = 0.03), LI (3.32; 95% CI: 1.52–7.28; *P* = 0.007) and TF (1.83; 95% CI: 0.85–3.93; P = 0.009).

**Conclusion:**

There was an inverse association between ∑ω-3/∑ω-6 ratio and general obesity and WC. We also found positive associations between abdominal obesity and AI and TF. Furthermore, TI and LI showed positive relationships with both general and abdominal obesity. Therefore, intake of dietary fatty acids in favor of higher ratios of ∑ω-3/∑ω-6 may be important in obesity prevention.

## Introduction

The prevalence of obesity and overweight are increasing globally and if the current trends are not controlled, 2.16 billion and 1.12 billion adults are likely to become overweight and obese, respectively, in the next decade [1, 2]. A previous review revealed alarming prevalence (43%) of overweight and obesity in Ghanaian adults [3]. According to epidemiological studies, the disease burden of obesity is enormous, it increases the risk of infertility in women [4, 5], metabolic syndrome [6, 7], cardiovascular diseases, type 2 diabetes mellitus (T2DM), chronic kidney disease [8], multiple cancers [9] and musculoskeletal complications [10, 11].

Although genetic factors play a role in the development of obesity [12, 13], the contributions of both total dietary fat [14, 15] intake and fat types [12, 13] have been suggested. However, the relationship between dietary fats and obesity is debatable [12, 14–21]. Specifically, positive [14, 15] and inverse [16] associations have been reported between total fat intake and obesity.

Moreover, previous studies suggested both positive [12, 14] and inverse [17] associations between dietary saturated fatty acids (SFAs) intake and weight gain. Furthermore, both positive [13, 14] and negative [18] relationships between monounsaturated fatty acids (MUFAs) intake and the chance of obesity have been found. Additionally, inverse [19, 20], null [14, 21] and positive [14] associations have been reported between polyunsaturated fatty acid (PUFA) consumption and the chance of obesity.

Typically, the fats and oils dietary pattern amongst Ghanaians is characterized by palm oil, shea butter and margarine [22, 23]. These fats have a high SFA content [24]. Palm oil, for example, contains approximately 50% SFA [24, 25] and Ghanaian shea butter has 45.9% stearic acid [26]. In two cross-sectional studies, this dietary pattern showed null [23] and unexpectedly inverse associations with general and abdominal obesity in Ghanaian adults [27].

Evidence from the above discrepant results may suggest that total fat intake and the absolute quantities of individual fatty acids consumed are not sensitive enough to reveal a consistent relationship between dietary fat and obesity. Therefore, the relative proportions of fatty acids instead of their absolute quantities in the diet may be an enhanced approach to investigate the relationship between dietary fat consumption and prevalence of obesity. In a previous study in French adults, low dietary PUFAs to SFAs ratio was found to accentuate the risk of being generally and abdominally obese [12]. In Africa, a prospective study found higher intake of unsaturated fatty acids to SFAs ratio and ∑ω-6/∑ω-3 ratio in normal weight compared with overweight/obese individuals with and without T2DM [18]. A major limitation in the African study was that the effect of dietary energy was not accounted for in the analysis [18], which could seriously confound the results [28, 29]. However, in Danish cohort, Lund et al. [19] did not find a significant association between ∑ω-3/∑ω-6 ratio with waist circumference and body mass index.

In addition to traditional approaches such as polyunsaturated fatty acids/saturated fatty acids ratio (PSR), and ∑ω-3/∑ω-6 ratios as stated in the preceding paragraph, a number of promising fat quality indices have been published to aid the evaluation of dietary fat quality (DFQ) in relation to health outcomes. First, Ulbricht and Southgate [30], in the early 90s, proposed the atherogenicity index (AI) and thrombogenicity index (TI), which consider the ratio of subclasses of SFAs to MUFAs and PUFAs. Subsequently, Santos-Silva et al. [31] published a fat quality index which considers the ratio between low cholesterol and high cholesterol raising fatty acids (h/H ratio) in foods. Later, Ding et al. [32] introduced the lipophilic index (LI) which has the ability to estimate the fluidity of biological specimens including cholesterol particles in the presence of fatty acids consumed or endogenously synthesized. Largely, previous work has focused on relationships between AI, TI, h/H or LI and incidence of gestational diabetes [33] and risk of myocardial infarction [34]. By considering the predictive effect of excess body weight gain on insulin sensitivity [35] and the importance of obesity in the development of many other chronic diseases including metabolic syndrome [6], it is imperative that scientists examine the association between the above fat quality indices and the odds of obesity. This may be a novel strategy for the development of nutrition policies that are beneficial for the prevention of obesity and excessive weight gain. It has not yet been established whether AI, TI, h/H ratio, ∑ω-3/∑ω-6 ratio, PSR or LI is associated with obesity/overweight in Ghana, a country faced with high prevalence of obesity and overweight [3]. Therefore, the purpose of this study was to investigate the relationship between general and abdominal obesity and six complementary fat quality indices (AI, TI, h/H ratio, ∑ω-3/∑ω-6 ratio, PSR, LI) and percentage of energy from total fat in women.

## Methods and materials

### Study design and population

This cross-sectional study was carried out among a randomly selected 295 women (aged 18–59 years) who live within Tamale Metropolis, Tamale, Ghana. Participation in this study was voluntary and participants also consented to the study without being coaxed. We collected data in August and September 2018. Based on our pre-defined criteria, potential participants of a reported history of myocardial infarction, renal disease or suffering from other major illnesses (diabetes, human immune deficiency virus, renal disease, cardiovascular disease**s** and malaria) were not allowed to take part in the study. Furthermore, the study excluded women with self-reported severe nausea and vomiting as well as those who were pregnant or lactating prior to recruitment. Moreover, we excluded women who were put on specific diet therapies or had been restricted from consuming a variety of foods. The study sample size was computed based on a hypothesized correlation coefficient of 0.2 between indices of fat quality and BMI, waist circumference (WC) and waist to height ratio (WHtR). Our pre-defined confidence level for the study was 95 and 80% power of test. Based on an established protocol for estimating sample sizes in cross-sectional studies by Rosner [36], a total of 195 women were first computed. In order to increase the power of the statistical results and to cater for non-response rate the 195 was multiplied by 1.5, which finally raised the final sample size to 295 subjects.

### Anthropometric assessment

Body weight was measured to the nearest 100 g using a Seca weighing scale (Seca weighing scale: Seca gmbh and co.kg; 22,089 Hamburg, Germany; Model: 8741321009; designed in Germany-made in China). Height measurement was by the use of United Nations Children Emergency Fund height board. The values were recorded to the nearest 0.1 cm. We calculated BMI as weight (in kilograms) divided by the height (in meters squared). BMI ≤24.9 kg/m^2^ was considered absence of general overweight and obesity. BMI values of 25–29.9 kg/m^2^ and BMI ≥ 30 kg/m^2^, defined overweight and general obesity, respectively [37]. The WC was measured based on World Health Organization guidelines thus, at the mid-point between the lower border of the rib cage and the iliac crest using a non-stretchable fiber-glass measuring tape. All WC values were recorded to the nearest 0.1 cm. Participants were dichotomized based on WC value [(normal: WC < 80 cm and abdominal obesity: WC ≥ 80 (cm)] [38]. We calculated WHtR by dividing WC (cm) by the measured height (cm). WHtR ≥0.5 was adopted for the presence of overweight and abdominal obesity for the purpose of uniformity regarding age differences whereas, WHtR ≤0.5 considered absence of overweight and abdominal obesity [39].

### Assessment of demographic and lifestyle factors

Information about demographic and lifestyle factors such as age, educational status, marital status, occupation, household size, household assets, parity, and physical activity were obtained using a structured questionnaire following face-to-face interviews. In order to estimate their economic status, we enumerated household assets for households that the selected study participants belong. Based on the list of items reported and the make-up of their rooms, we calculated a summary value as a proxy for economic status. Physical activity level of participants was measured using the International Physical Activity Questionnaire short form [40]. The reliability and validity of these questionnaires were assessed across 12 countries and the results showed they can be applied in many settings and in different languages [41]. With these questionnaires, we asked questions about the time and the number of days within the previous seven (7) days a person spent on walking, doing moderate-intensity activity and/or doing vigorous-intensity activities. The overall physical activity level in metabolic equivalent minutes per week (MET-minutes/week) was calculated by summing the totals for walking activity, moderate activity and vigorous activity in MET-minutes/week scores.

### Dietary intake

All dietary data were obtained based on repeated non-consecutive 2-day 24-h dietary recalls. We used real food items, food models and standard kitchen weighing equipment to guide food portion size estimation. We deployed a trained caterer for the interviews. Absolute quantities of dietary fatty acids were determined using standard portion sizes of food items from the United States Department of Agriculture Food Composition Databases [42] due to a lack of local database on this issue. For the same reason, we obtained melting points of dietary fatty acids from the Japanese Lipid Bank database [43]. The mean values of daily intake of total fat, fatty acids, and energy for the two days were used in the analysis.

We calculated energy percentage from fat (TF) from dividing average dietary energy obtained from fat (kcals) by average daily total energy intake, and finally expressed the results as 100%. Also, we calculated DFQ indices from established protocols as detailed below.

Polyunsaturated fatty acids/saturated fatty acids ratio (PSR) = PUFAs: SFAs ratio (1) [30].

Atherogenicity index (AI) = (l2:0 + (4 x l4:0) + 16:0) / (∑ω-6 PUFA +∑ ω-3 PUFA + ∑MUFA ω-9) (2) [30]; where 12:0 = lauric acid,14:0 = myristic acid, 16:0 = palmitic acid, ∑ω-6 PUFA = sum of omega-6 polyunsaturated fatty acids, ∑ω-3 PUFA = sum of omega-3 polyunsaturated fatty acids, and ∑MUFA ω-9 = sum of omega-9 monounsaturated fatty acids.

Thrombogenic index (TI) = [(14:0+ 16:0 + 18:0) / [(0.5∑MUFA)] + (0.5∑ω-6 PUFA) + (3 ∑ω-3 PUFA) + (∑ω-3 PUFA / ∑ω-6 PUFA)] (3) [30]; where 14:0 = myristic acid, 16:0 = palmitic acid, 18:0 = stearic acid, MUFA = monounsaturated fatty acids, ∑ω-6 PUFA = sum of omega-6 polyunsaturated fatty acids, ∑ω-3 PUFA = sum of omega-3 polyunsaturated fatty acids.

hypo- and hypercholesterolemic fatty acids ratio (h/H) = (18: 1 ω-9 + 18: 2 ω-6 + 20: 4 ω-6 + 18: 3 ω-3 + 20: 5 ω-3 + 22: 5 ω-3 + 22: 6 ω-3 /(14: 0 + 16: 0) (4) [31]; where 18:1 ω-9 = oleic acid, 18:2 ω-6 = linoleic acid, 20:4 ω-6 = arachidonic acid, 18:3 ω-3 = alpha-linolenic acid, 20:5 ω-3 = eicosapentaenoic acid, 22:5 ω-3 = docosapentaenoic acid, 22:6 ω-3 = docosahexaenoic acid, 14:0 = myristic acid, 16:0 = palmitic acid, h/H = hypo- and hypercholesterolemic fatty acids ratio.

∑ω-3 / ∑ω-6 ratio (5) [30]; where ∑ω-3 = sum of omega-3 polyunsaturated fatty acids, ∑ω-6 = sum of omega-6 polyunsaturated fatty acids.

The lipophilic index of dietary intake was computed by multiplying the intake of each fatty acid (in grams) by its specific melting point (°C), adding the products, and then dividing by the total of fatty acid intake (in grams) [34].

Dietary lipophilic index (LI) = ∑[Fatty acid (g) i × Melting point (°C)i] / ∑Fatty acid (g) i (6) [32]; where LI = dietary lipophilic index.

### Statistical analysis

All data were checked for compliance with the selected statistical techniques. The Kolmogorov–Smirnov test was used to evaluate the normality of the data distribution. To use physical activity as a continuous variable in our regression models, it was first transformed (natural logarithm) because it was positively skewed. Based on our predefined dietary energy cut-off values, subjects were excluded whose reported average dietary energy intake levels were below < 500 kcal/d or above > 3500 kcal/d [44]. Out of the 295 subjects sampled for the study, we excluded 18 participants because their reported dietary energy intakes were outside the cut off values.

The DFQ indices were energy-adjusted using the residual method [45]. Energy-adjusted AI, TI, h/H ratio, ∑ω-3/∑ω-6 ratio, PSR, LI, and TF were used to classify participants into quintiles (Qs). Participants were also dichotomized as those with and without overweight and obesity based on BMI, WC, and WHtR. The means ± standard deviations (SDs) for socio-demographic factors were reported for continuous variables by the use of independent-sample t-test in participants classified as having obesity/overweight and those without obesity/overweight whereas frequencies and percentages were presented for categorical variables using a Chi-square test. Additionally, we compared means and standard deviations for BMI, WC, and WHtR across the Qs of DFQ indices, using a One-way analysis of variance (ANOVA). Binary logistic regression was used to determine the odds ratios (ORs) and 95% confidence intervals (CIs) for the presence of general and abdominal obesity across Qs of DFQ indices in unadjusted and multivariable-adjusted models. The possible effects of all potential confounding variables that showed a significant relationship with measures of obesity and overweight were adjusted. In this context, age (continuous); asset score (continuous); physical activity level (natural log-transformed); dietary energy/kcal (continuous); woman education (categorical); husband education (categorical); occupation woman (categorical); occupation husband (categorical); marital status (categorical) were entered into the adjusted regression models. The IBM Statistical Package for Social Sciences (version 24; SPSS Inc.) was used for all statistical analyses. We considered *P* < 0.05 to be statistically significant for all analyses.

## Results

Of the 277 women assessed, 22.7% were overweight according to BMI indicator, 9.4% had general obesity based on BMI, and 32.5 and 39.4% had abdominal obesity based on WC and high WHtR, respectively. Characteristics of study participants based on obesity/overweight status are presented in Table 1. Participants who had general obesity (P for trend = 0.01), abdominal obesity based on WC (P for trend < 0.001), and high WHtR (P for trend < 0.001) were older, when compared with those without obesity. Similarly, the highest average asset score was found in participants classified as having general obesity (P for trend = 0.003), abdominal obesity based on both WC (P for trend < 0.001) and high WHtR (P for trend = 0.03) in comparison with their respective counterparts without obesity.

**Table 1:** Characteristic of study participants according to subjects with obesity/overweight and without obesity/overweight

Variable	BMI (Kg/m^2^)		P	WC (cm)		P	WHtR		P
	≥25	≤24.9		≥80	< 80		≥0.50	< 0.50	
Age (years)	38.1 ± 10.7	34.6 ± 11.2	0.01	39.5 ± 10.2	33.9 ± 11.1	< 0.001	39.3 ± 10.7	33.3 ± 10.7	< 0.001
Parity	3.6 ± 2.1	2.9 ± 1.9	0.008	3.9 ± 2.1	2.8 ± 1.9	< 0.001	3.9 ± 2.0	2.6 ± 1.9	< 0.001
Household Size	9.6 ± 5.1	10.8 ± 6.3	0.09	9.8 ± 5.3	10.8 ± 6.3	0.2	9.9 ± 5.6	10.8 ± 6.1	0.3
Asset score^c^	54.4 ± 4.4	52.8 ± 3.9	0.003	54.7 ± 4.7	52.6 ± 3.7	< 0.001	53.9 ± 4.5	52.8 ± 3.8	0.03
Energy intake (kcal/day)	2154.9 ± 663.7	2048.1 ± 694.7	0.2	2303.3 ± 677.5	1976.1 ± 665.4	< 0.001	2223.9 ± 672.8	1990.5 ± 679.8	0.005
Physical Activity^a^									
Low	35(59.3)	24(40.7)		38(64.4)	21(35.6)		46(78.0)	13(22.0)	
Moderate	42(26.9)	114(73.1)	< 0.001	42(26.9)	114(73.1)	< 0.001	52(33.3)	104(66.7)	< 0.001
High	12(19.4)	50(80.6)		10(16.1)	52(83.9)		11(17.7)	51(82.3)	
Ethnicity									
Dagomba	68(30.9)	152(69.1)	0.2	68(30.9)	152(69.1)	0.1	84(38.2)	136(61.8)	0.4
Minority^b^	21(36.8)	36(63.2)		22(38.6)	35(61.4)		25(43.9)	32(56.1)	
Marital Status									
Married	83(33.6)	164(66.4)	0.09	84(34.0)	163(66.0)	0.08	103(41.7)	144(58.3)	0.02
Single	6(20.0)	24(80.0)		6(20.0)	24(80.0)		6(20.0)	24(80.0)	
Woman Education									
No formal Education	59(35.5)	107(64.5)		64(38.6)	102(61.4)		74(44.6)	92(55.4)	
Primary/Middle/JHS	12(21.1)	45(78.9)	0.01	11(19.3)	46(80.7)	0.02	13(22.8)	44(77.2)	0.01
SHS/Tertiary	18(33.3)	36(66.7)		15(27.8)	39(72.2)		22(40.7)	32(59.3)	
Husband Education									
No formal Education	46(35.4)	84(64.6)		45(34.6)	85(65.4)		58(44.6)	72(55.4)	
Primary/Middle/JHS	6(10.0)	54(90.0)	< 0.001	8(13.3)	52(86.7)	< 0.001	8(13.3)	52(86.7)	< 0.001
SHS/Tertiary	32(56.1)	25(43.9)		32(56.1)	25(43.9)		38(66.7)	19(33.3)	
Occupation of woman									
Farmer	16(38.1)	26(61.9)		12(28.6)	99(71.4)		15(35.7)	27(64.3)	
Trader	48(31.6)	104(68.4)		53(34.9)	99(65.1)		68(44.7)	84(55.3)	
Salary/Service Sector	9(29.0)	22(71.0)	0.8	6(19.4)	25(80.6)	0.3	8(25.8)	23(74.2)	0.1
Housewife	16(30.8)	36(69.2)		19(36.5)	33(63.5)		18(34.6)	34(65.4)	
Occupation of husband									
Farmer	29(33.0)	59(67.0)		26(29.5)	62(70.5)		33(37.5)	55(62.5)	
Salary/Service Sector	21(28.4)	43(71.6)	0.3	21(28.4)	53(71.6)	0.07	27(36.5)	47(63.5)	0.1
Trader	33(38.8)	52(61.2)		37(43.5)	48(56.5)		43(50.6)	42(49.4)	

Data are presented as mean ± standard deviation and number (%)

^a^; Low (<600MET-minuts/week); Moderate (≥600 but < 3000 MET- minutes/week); High (≥3000 MET-minutes/weeks), p-trend (p); Test of trend was conducted using ANOVA with contrast function for continuous variables and χ2 test for categorical variables. P < 0·05 was considered as statistically significant

^b^Gonja, gurusi, zambarama, Moshi, JHS (Junior High School); SHS (Senior High School, g/d: gram per day

^c^An ad hoc index of household asset score was computed from basically number of assets possessed from a list of items provided. A score of 1 point was given for an item owned. As for the main source of cooking energy, drinking water and toilet facility, 1 point was given for unimproved source and 2 points awarded for improved sources. The total score range was from 37 to 74 with higher scores suggesting enhanced economic strength. *WC* Waist circumference, *BMI* Body Mass Index, *WHtR* Waist to Height Ratio

Anthropometric measures according to DFQ indices are presented in Table 2. According to the findings, mean BMI decreased significantly from the lowest to the highest Q of h/H ratio (p-trend = 0.04). However, this relationship attenuated after adjustment for age, asset score, physical activity level, total dietary energy, woman education, husband education, occupation woman, occupation husband and marital status (p-trend = 0.6). In the context of TI Qs, the mean BMI (p-trend = 0.04) and WC (p-trend = 0.03) increased significantly from the lowest to the highest Q. These patterns in means weakened after adjustment for the covariates for both BMI (p-trend = 0.06) and WC (p-trend = 0.07). Moreover, after these adjustments for covariates, mean WHtR also increased significantly from the lowest to the highest Q of TI (p-trend = 0.03). Additionally, we observed that mean BMI significantly increased (p-trend = 0.04) from the lowest to the highest Q of LI. This trend was still significant even after adjustment for potential confounding factors (p-trend = 0.04). Although an increase in mean WHtR across the lowest to the highest Q of LI was found not to be significant (p-trend = 0.1), in the unadjusted model, it was significant in the multivariable-adjusted model (p-trend = 0.02). Means of WC and WHtR did not show appreciable patterns with PSR, AI, ∑ω-3/∑ω-6 ratio and TF.

**Table 2:** Anthropometric measures according to quintiles (Q) of fat quality indices

Variable	BMI(Kg/m^2^)			WC (cm)			WHtR		
	Mean ± SD	P1	P2	Mean ± SD	P1	P2	Mean ± SD	P1	P2
**PSR**									
Q1 (55)	25.2 ± 5.2			81.6 ± 10.2			0.50 ± 0.06		
Q2 (56)	22.0 ± 5.2			72.7 ± 13.5			0.46 ± 0.08		
Q3 (55)	22.5 ± 3.6	0.6	0.1	75.7 ± 7.4	0.9	0.06	0.47 ± 0.05	0.9	0.1
Q4 (56)	24.5 ± 4.1			80.0 ± 9.2			0.50 ± 0.06		
Q5 (55)	23.5 ± 4.2			77.9 ± 9.2			0.48 ± 0.06		
∑**ω-3/**∑**ω-6**									
Q1 (55)	21.6 ± 4.4			72.8 ± 9.9			0.46 ± 0.06		
Q2 (56)	23.9 ± 4.6			79.3 ± 10.4			0.50 ± 0.07		
Q3 (55)	24.3 ± 4.9	0.09	0.057	78.4 ± 10.0	0.1	0.03	0.48 ± 0.06	0.07	0.058
Q4 (56)	25.4 ± 4.2			83.1 ± 8.8			0.52 ± 0.06		
Q5 (55)	22.5 ± 4.4			74.3 ± 10.3			0.47 ± 0.07		
**h/H**									
Q1 (55)	25.5 ± 5.5			81.4 ± 12.4			0.51 ± 0.07		
2 (56)	21.9 ± 4.1			73.0 ± 8.9			0.46 ± 0.06		
Q3 (55)	24.1 ± 4.6	0.04	0.6	78.9 ± 11.3	0.4	0.7	0.49 ± 0.07	0.2	0.7
Q4 (56)	23.4 ± 4.2			77.3 ± 8.6			0.49 ± 0.06		
Q5 (55)	22.8 ± 3/9			77.4 ± 9.5			0.48 ± 0.06		
**AI**									
Q1 (55)	22.8 ± 4.1			76.8 ± 9.9			0.47 ± 0.06		
Q2 (56)	24.2 ± 4.4			79.2 ± 8.4			0.49 ± 0.06		
Q3 (55)	23.5 ± 3.6	0.2	0.4	78.1 ± 7.9	0.5	0.3	0.48 ± 0.05	0.4	0.2
Q4 (56)	21.7 ± 4.9			71.6 ± 12.4			0.45 ± 0.08		
Q5 (55)	25.4 ± 5.3			82.2 ± 10.7			0.51 ± 0.07		
**TI**									
Q1 (55)	22.4 ± 3.1			74.7 ± 7.1			0.47 ± 0.05		
Q2 (56)	24.5 ± 4.8			80.5 ± 10.7			0.49 ± 0.06		
Q3 (55)	22.9 ± 3.9	0.04	0.06	75.8 ± 9.0	0.03	0.07	0.48 ± 0.06	0.06	0.03
Q4 (56)	22.3 ± 4.9			75.1 ± 12.4			0.47 ± 0.07		
Q5 (55)	25.5 ± 5.3			82.0 ± 10.8			0.51 ± 0.08		
**LI**									
Q1 (55)	22.9 ± 3.8			77.6 ± 9.3			0.48 ± 0.05		
Q2 (56)	23.9 ± 4.3			77.4 ± 9.2			0.48 ± 0.06		
Q3 (55)	23.2 ± 4.5	0.03	0.04	77.1 ± 9.3	0.2	0.07	0.48 ± 0.06	0.1	0.02
Q4 (56)	21.9 ± 4.7			73.1 ± 12.4			0.46 ± 0.07		
Q5 (55)	26.0 ± 5.1			82.7 ± 10.1			0.52 ± 0.06		
**TF**									
Q1 (55)	24.0 ± 5.4			79.8 ± 12.7			0.49 ± 0.08		
Q2 (56)	23.7 ± 4.2			79.0 ± 9.1			0.49 ± 0.05		
Q3 (55)	23.3 ± 3.8	0.7	0.1	77.2 ± 8.2	0.06	0.03	0.48 ± 0.05	0.1	0.06
Q4 (56)	22.2 ± 3.9			73.5 ± 9.6			0.46 ± 0.06		
Q5 (55)	24.4 ± 5.3			78.4 ± 11.5			0.48 ± 0.07		

P for trend (P1; unadjusted model test of trend using ANOVA with contrast function, P2; Significance level of test for trend association in adjusted linear regression model with adjustment for age (continuous); asset score (continuous); physical activity level (natural log transformed); dietary energy/kcal (continuous); woman education (categorical); husband education (categorical); occupation woman (categorical); occupation husband (categorical); marital status (categorical). In all multiple regression models, dummy variables were created for categorical covariates. To analyze the linear trends in the adjusted models, the median values of PSR, ∑ω-3/∑ω-6, h/H, AI, TI, LI and TF were imputed for each quintile and the new variables were treated as continuous variables in linear regression model. TF; percentage of energy obtained from dietary fat. P < 0·05 was considered statistically significant. *WC* Waist circumference, *BMI* Body Mass Index, *WHtR* Waist to Height Ratio, *ANOVA* One-Way Analysis of Variance, *PSR* polyunsaturated to saturated fatty acids ratio, ∑ω-3/∑ω-6; omega-3 to omega-6 fatty acids ratio, *h/H* hypo and hypercholesterolemic fatty acids ratio, *AI* Atherogenicity index, *TI* Thrombogenic index, *LI* Dietary lipophilic index, *TF* Percentage of energy from total fat

The ORs of obesity according to quintiles (Q) of fat types are presented in Table 3. Logistic regression analysis showed a positive association between total SFA and general obesity (OR_quintiles 5 vs.1_ 2.16; 95% CI 0.99–4.70; p-trend = 0.02). However, after adjustment for age, asset score, physical activity level, total dietary energy, woman education, husband education, occupation woman, occupation husband, and marital status, the relationship was not remained significant (OR_quintiles 5 vs.1_ 1.41; 95% CI 0.53–3.78; p-trend = 0.2). Additionally, SFA was positively associated with WC in both the unadjusted (OR_quintiles 5 vs.1_ 2.49; 95% CI 1.14–5.44; p-trend = 0.002) and the adjusted (OR_quintiles 5 vs.1_ 1.07; 95% CI 0.37–3.12; p-trend = 0.02) models. Also, SFA had a significant positive association with WHtR in the unadjusted model only (OR_quintiles 5 vs.1_ 2.64; 95% CI 1.22–5.71; p-trend = 0.01). In the unadjusted model, PUFA showed a positive association with WC (OR_quintiles 5 vs.1_ 1.29; 95% CI 1.19–6.10; p-trend = 0.01). However, in the adjusted model, this relationship was not significant. The ∑ω-3 PUFAs and the ∑ω-6 PUFAs did not show any relationship with obesity.

**Table 3:** Odds ratio (95% CI) of general and abdominal obesity according to quintiles (Q) of fat types

variable	GO (no/yes)			WC ≥ 80 cm (no/yes)			WHtR≥0.50(no/yes)		
	OR (95%CI)	P1	P2	OR (95%CI)	P1	P2	OR (95%CI)	P1	P2
**Total SFA**									
Q1 (55)	**Reference**								
Q2 (56)	1.45(0.66–3.17)			1.15(0.52–2.54)			1.64(0.76–3.53)		
	1.28(0.50–3.29)			0.48(0.16–1.39)			1.17(0.44–3.11)		
Q3 (55)	0.76(0.33–1.75)	0.02	0.2	0.76(0.33–1.76)	0.002	0.02	0.77(0.35–1.74)	0.01	0.09
	0.56(0.18–1.71)			0.14(0.04–0.51)			0.29(0.09–0.97)		
Q4 (56)	0.43(0.17–1.07)			0.55(0.23–1.31)			0.75(0.34–1.69)		
	0.30(0.09–1.03)			0.08(0.02–0.36)			0.39(0.12–1.32)		
Q5 (55)	2.16(0.99–4.70)			2.49(1.14–5.44)			2.64(1.22–5.71)		
	1.41(0.53–3.78)			1.07(0.37–3.12)			1.42(0.50–3.99)		
Total PUFA									
Q1 (55)	**Reference**								
Q2 (56)	1.26(0.56–2.86)			1.08(0.45–2.56)			1.46(0.66–3.24)		
	1.01(0.38–2.72)			0.76(0.25–0.27)			1.35(0.47–3.80)		
Q3 (55)	1.30(0.57–2.94)	0.7	0.4	1.45(0.62–3.37)	0.01	0.5	1.63(0.74–3.59)	0.050	0.7
	0.77(0.26–2.82)			0.76(0.24–2.42)			0.99(0.32–3.06)		
Q4 (56)	1.73(0.78–3.84)			1.94(0.85–4.42)			1.83(0.83–4.01)		
	1.09(0.35–3.35)			1.22(0.37–4.05)			1.20(0.37–3.88)		
Q5 (55)	1.09(0.48–2.51)			2.69(1.19–6.10)			2.19(0.25–1.18)		
	0.50(0.12–2.00)			1.29(0.31–5.40)			1.41(0.34–5.81)		
∑ω-3 **PUFA**									
Q1 (55)	**Reference**								
Q2 (39)	0.88(0.34–2.30)			1.27(0.50–3.23)			0.96(0.39–2.38)		
	0.73(0.23–2.35)			1.68(0.51–5.61)			0.96(0.29–3.17)		
Q3 (71)	2.14(0.99–4.62)	0.1	0.1	2.10(0.96–4.60)	0.1	0.3	1.68(0.80–3.56)	0.6	0.2
	3.00(1.14–7.94)			3.23(1.13–9.24)			2.63(0.94–7.41)		
Q4 (52)	2.93(1.30–6.66)			4.07(1.78–9.33)			4.23(1.88–9.52)		
	2.87(1.05–7.81)			5.12(1.78–14.71)			6.70(2.30–19.52)		
Q5 (60)	0.59(0.24–1.46)			0.57(0.22–1.46)			1.22(0.55–2.69)		
	0.60(0.20–1.80)			0.84(0.26–2.76)			2.10(0.71–6.20)		
∑ω-6 **PUFA**									
Q1 (58)	**Reference**								
Q2 (47)	1.26(0.56–2.84)			0.69(0.31–1.58)			1.02(0.46–2.24)		
	1.48(0.56–3.93)			0.51(0.18–1.48)			0.94(0.34–2.61)		
Q3 (61)	1.17(0.54–2.51)	0.2	0.8	0.74(0.35–1.58)	0.3	0.5	0.92(0.44–1.94)	0.08	0.2
	1.5(0.61–3.74)			0.84(0.33–2.17)			1.26(0.49–3.21)		
Q4 (56)	0.48(0.20–1.17)			0.45(0.20–1.02)			0.84(0.39–1.81)		
	0.37(0.13–1.04)			0.29(0.10–0.85)			0.64(0.24–1.7)		
Q5 (55)	1.60(0.74–3.46)			1.18(0.55–2.50)			1.70(0.80–3.59)		
	1.27(0.51–3.16)			1.01(0.39–2.63)			1.65(0.64–4.28)		

P1: unadjusted; P2: adjusted for age (continuous); asset score (continuous); physical activity level (natural log transformed); dietary energy/kcal (continuous); woman education (categorical); husband education (categorical); occupation woman (categorical); occupation husband (categorical); marital status (categorical). In the multiple regression models, dummy variables were created for categorical covariates. TF; percentage of energy obtained from dietary fat. P; Test of linear trends across quintiles of SFA, PUFAs, ∑ω-3, and ∑ω-6 were calculated for the models assessing chance of overweight/obesity as measured by body mass index, waist circumference and waist circumference to height ratio. To analyze these linear trends, the median values of total SFA, PUFAs, ∑ω-3, and ∑ω-6 were imputed for each quintile and the new variables were treated as continuous variables. All tests statistics are considered significant for P < 0.05, *OR* Odds ratio, *CI* Confidence Interval, *GO* General obesity, *WC* waist circumference, *WHtR* waist to height ratio, *SFA* Saturated fatty acids, *PUFA* Polyunsaturated fatty acids, ∑ω-3; Summation of omega-3 polyunsaturated fatty acids, ∑ω-6; Summation of omega-6 polyunsaturated fatty acids

Logistic regression analysis showed an inverse association between ∑ω-3/∑ω-6 ratio and general obesity (OR_quintiles 5 vs.1_ 0.63; 95% CI 0.24–1.63; p-trend = 0.01). This relationship attenuated (OR_quintiles 5 vs.1_ 0.78; 95% CI 0.22–2.81; p-trend = 0.005) upon adjustment for the effects of age, asset score, physical activity level, total dietary energy, woman education, husband education, occupation woman, occupation husband, and marital status. Although in the unadjusted model, the analysis suggested a null association between ∑ω-3/∑ω-6 ratio and abdominal obesity defined by WC, in the adjusted model, there was a significant inverse association when the highest Q was compared with the lowest Q (OR_quintiles 5 vs.1_ 0.73; 95% CI 0.18–3.02; p-trend = 0.02). Also, a significant inverse association was seen between h/H ratio and high WHtR when the highest Q of h/H ratio was compared with the lowest Q (OR_quintiles 5 vs.1_ 0.29; 95% CI 0.13–0.65; p-trend = 0.03). Even after adjustment for covariates, this relationship remained significant. No relationship was found between the h/H ratio and both general obesity and high WC. Moreover, PSR was not associated with obesity parameters.

In regard of the AI, a significant increase in the chance of general obesity (OR_quintiles 5 vs.1_ 1.98, 95% CI 0.91–4.29; p-trend = 0.03) among participants in the highest Q of AI emerged when compared with the lowest Q, however, this attenuated after adjustment for multiple confounders. Similarly, a significant increase in OR for abdominal obesity based on WC (OR_quintiles 5 vs.1_ 1.24, 95% CI 0.56–2.74; p-trend = 0.008) and WHtR (OR_quintiles 5 vs.1_ 2.89, 95% CI 1.32–6.31; p-trend = 0.01) was found among participants included in the highest Q of AI and remained significant even after adjustment for multiple confounders. When analysis was carried out, in the highest quintile of TI compared with the lowest quintile, the OR increased for general obesity (OR_quintiles 5 vs.1_ 4.14, 95% CI 1.78–9.66; p-trend = 0.003), abdominal obesity based on both WC (OR_quintiles 5 vs.1_ 4.14, 95% CI 1.78–9.66; p-trend = 0.004) and high WHtR (OR_quintiles 5 vs.1_ 2.65, 95% CI 1.22–5.76; p-trend = 0.02) criteria. These observations remained unchanged upon multiple variable adjustments. Additionally, significant positive associations were observed between LI and general obesity (OR_quintiles 5 vs.1_ 2.49, 95% CI 1.14–5.43; p-trend = 0.006) and abdominal obesity as defined by both WC (OR_quintiles 5 vs.1_ 2.11, 95% CI 0.98–4.55; p-trend = 0.01) and high WHtR (OR_quintiles 5 vs.1_ 3.32, 95% CI 1.52–7.28; p-trend = 0.004) and were still significant even after adjustment for covariates. In the unadjusted model, we found non-significant positive relationship between TF and abdominal obesity based on both WC and WHtR definitions, however, after multiple variable adjustment, we found significant positive associations between TF and abdominal obesity defined by both WC (OR_quintiles 5 vs.1_ 1.59; 95% CI, 0.73–3.46; P-trend = 0.003) and high WHtR (OR_quintiles 5 vs.1_ 1.83; 95% CI 0.85–3.93; p-trend = 0.009) (Table 4).

**Table 4:** odds ratio (95% CI) of general and abdominal obesity according to quintiles (Q) of fat quality indices

variable	GO (no/yes)			WC ≥ 80 cm (no/yes)			WHtR≥0.50(no/yes)		
	OR (95%CI)	P1	P2	OR (95%CI)	P1	P2	OR (95%CI)	P1	P2
**PSR**									
Q1(55)	**Reference**								
Q2 (56)	0.25(0.11–0.59)			0.15(0.06–0.37)			0.26(0.12–0.58)		
	0.26(0.09–0.72)			0.08(0.02–0.29)			0.24(0.08–0.69)		
Q3	0.26(0.11–0.60)	0.3	0.7	2.00(0.08–0.47)	0.3	0.6	0.44(0.21–0.95)	0.2	0.3
	0.20(0.07–0.54)			0.07(0.02–0.24)			0.30(0.11–0.80)		
Q4	0.62(0.29–1.33)			0.72(0.34–1.53)			0.67(0.32–1.42)		
	0.75(0.29–1.89)			0.81(0.30–2.21)			0.74(0.28–1.92)		
Q5	0.55(0.25–1.18)			0.44(0.20–0.95)			0.37(0.17–0.82)		
	0.73(0.28–1.90)			0.49(0.18–1.40)			0.42(0.15–1.16)		
∑**ω-3/**∑**ω-6**									
Q1 (55)	**Reference**								
Q2 (56)	2.42(1.07–5.48)			3.13(1.31–7.46)			2.68(1.17–6.16)		
	3.81(1.22–11.87)			3.11(0.91–10.70)			4.42(1.31–14.97)		
Q3 (55)	1.65(0.72–3.81)	0.01	0.005	2.13(0.87–5.16)	0.08	0.02	2.15(0.93–4.97)	0.9	0.8
	3.03(0.82–11.14)			1.89(0.46–7.74)			5.33(1.34–21.12)		
Q4 (56)	2.50(1.10–5.67)			5.01(2.11–11.93)			5.37(2.32–12.41)		
	3.39(0.84–13.68)			3.14(0.70–14.15)			9.28(2.10–40.99)		
Q5 (55)	0.63(0.24–1.63)			1.00(0.37–2.63)			1.89(0.81–4.41)		
	0.79(0.22–2.81)			0.73(0.18–3.02)			3.59(0.99–12.99)		
**h/H**									
Q1 (55)	**Reference**								
Q2 (56)	0.13(0.05–0.35)			0.13(0.05–0.35)			0.28(0.13–0.63)		
	0.13(0.04–0.40)			0.11(0.03–0.35)			0.27(0.10–0.75)		
Q3 (55)	0.50(0.23–1.09)	0.4	0.4	0.50(0.23–1.09)	0.6	0.3	0.51(0.24–1.10)	0.03	0.01
	0.59(0.23–1.49)			0.46(0.17–1.24)			0.53(0.20–1.42)		
Q4 (56)	0.45(0.21–0.98)			0.45(0.21–0.98)			0.46(0.21–0.99)		
	0.55(0.22–1.40)			0.31(0.11–0.87)			0.39(0.14–1.06)		
Q5 (55)	0.43(0.19–0.94)			0.46(0.21–1.01)			0.29(0.13–0.65)		
	0.38(0.15–0.99)			0.39(0.14–1.06)			0.20(0.07–0.56)		
**AI**									
Q1 (55)	**Reference**								
Q2 (56)	1.23(0.57–2.69)			1.24(0.56–2.74)			1.68(0.77–3.65)		
	1.89(0.68–5.14)			1.21(0.41–3.54)			2.55(0.88–7.46)		
Q3 (55)	0.70(0.31–1.61)	0.03	0.1	0.92(0.41–2.07)	0.008	0.01	1.61(0.73–3.52)	0.01	0.04
	0.20(0.37–2.68)			1.12(0.40–3.15)			2.58(0.94–7.14)		
Q4 (56)	0.39(0.16–0.97)			0.37(0.15–0.96)			0.75(0.32–1.71)		
	0.40(0.13–1.19)			0.27(0.08–0.93)			0.99(0.33–3.00)		
Q5 (55)	1.98(0.91–4.29)			1.24(0.56–2.74)			2.89(1.32–6.31)		
	2.43(0.92–6.42)			2.30(1.07–8.38)			4.17(1.45–11.99)		
**TI**									
Q1 (55)	**Reference**								
Q2 (56)	2.58(1.10–6.06)			2.78(1.19–6.51)			1.54(0.71–3.33)		
	2.76(1.07–7.16)			(2.580.96–6.93)			1.71(0.68–4.31)		
Q3 (55)	1.64(0.68–3.95)	0.003	0.01	1.50(0.61–3.64)	0.004	0.009	1.17(0.53–2.58)	0.02	0.03
	1.69(0.62–4.65)			1.24(0.41–3.76)			1.03(0.38–2.79)		
Q4 (56)	1.09(0.43–2.73)			1.20(0.48–2.99)			0.82(0.36–1.84)		
	1.44(0.48–4.29)			1.10(0.35–3.48)			0.82(0.29–2.38)		
Q5 (55)	4.14(1.78–9.66)			4.14(1.78–9.66)			2.65(1.22–5.76)		
	4.93(1.74–13.95)			4.85(1.62–14.49)			3.01(1.08–8.39)		
**LI**									
Q1 (55)	**Reference**								
Q2 (56)	0.97(0.43–2.18)			0.89(0.40–1.97)			1.54(0.71–3.33)		
	1.36(0.54–3.44)			1.71(0.64–4.52)			2.74(1.04–7.20)		
Q3 (55)	0.91(0.40–2.07)	0.006	0.01	0.71(0.31–1.60)	0.01	0.02	1.17(0.53–2.58)	0.004	0.007
	1.30(0.51–3.32)			0.86(0.31–2.36)			1.90(0.72–5.07)		
Q4 (56)	0.48(0.19–1.18)			0.36(0.14–0.89)			0.62(0.26–1.43)		
	0.60(0.21–1.71)			0.28(0.09–0.92)			0.60(0.20–1.85)		
Q5 (55)	2.49(1.14–5.43)			2.11(0.98–4.55)			3.32(1.52–7.28)		
	3.26(1.27–8.39)			3.26(1.20–8.83)			5.81(2.06–16.41)		
**PSR**									
Q1(55)	**Reference**								
Q2 (56)	1.35(0.61–3.01)			0.97(0.44–2.16)			1.32(0.61–2.85)		
	1.48(0.57–3.84)			1.22(0.44–3.36)			1.20(0.74–5.39)		
Q3	0.91(0.40–2.10)	0.08	0.06	1.09(0.49–2.39)	0.2	0.003	1.08(0.50–2.37)	0.1	0.009
	0.97(0.36–2.64)			1.58(0.56–4.43)			1.55(0.56–4.33)		
Q4	0.73(0.32–1.73)			0.50(0.21–1.20)			1.05(0.48–2.30)		
	0.75(0.26–2.15)			0.75(0.24–2.38)			1.87(0.63–5.54)		
Q5	2.03(0.92–4.47)			1.59(0.73–3.46)			1.83(0.85–3.93)		
	2.54(0.91–7.04)			4.71(1.57–14.10)			4.44(1.52–13.00)		

P1: unadjusted; P2: adjusted for age (continuous); asset score (continuous); physical activity level (natural log transformed); dietary energy/kcal (continuous); woman education (categorical); husband education (categorical); occupation woman (categorical); occupation husband (categorical); marital status (categorical). In the multiple regression models, dummy variables were created for categorical covariates. P; Tests of linear trend across increasing PSR, ∑ω-3/∑ω-6, h/H, AI, TI, LI and TF were calculated for the models assessing chance of overweight/obesity as measured by body mass index, waist circumference and waist circumference to height ratio. To analyze these linear trends, the median values of PSR, ∑ω-3/∑ω-6, h/H, AI, TI, LI and TF were imputed for each quintile and the new variables were treated as continuous variables. All tests statistics are considered significant for P < 0.05, *OR* Odds ratio, *CI* Confidence Interval, *GO* General obesity (BMI ≥ 25), *WC* waist circumference, *WHtR* waist to height ratio, *PSR* polyunsaturated to saturated fatty acids ratio, ∑ω-3/∑ω-6; omega-3 to omega-6 fatty acids ratio, *h/H* hypo and hypercholesterolemic ratio, *AI* Atherogenicity index, *TI* Thrombogenic index, *LI* Dietary lipophilic index, *TF* Percentage of energy from total fat

## Discussion

The present study evaluated the relationship between general and abdominal obesity, fat types, and indices of dietary fat quality amongst Ghanaian women. A separate assessment of the independent relationships between fatty acid types and obesity have largely failed to show significant associations, except that the total SFA intake only had a positive relationship with WC. In contrast, when the fatty acids were considered as indices, independent of other covariates, general obesity was inversely associated with ∑ω-3/∑ω-6 ratio and positively with TI and LI. Moreover, abdominal obesity had a positive association with AI, TI, LI, and TF. These associations support the proposition that compared with fat types, the dietary fatty acid indices may show a better relationship with health outcomes [30, 32].

The intake of absolute amounts of dietary fatty acids appears not to have a consistent relationship with obesity. For instance, a previous observational study found no association between adiposity and the ω-3 PUFA and SFA intake [14]. Also, another observational study found an inverse association between dietary SFA and WC after adjustment for age. However, this association was no longer significant after an additional adjustment for BMI [46].

Unlike the present study, in which the prevalence of obesity was evaluated in association with dietary fatty acids in the form of indices, most previous studies in Ghana only assessed associations between type of cooking fats and oils [23, 47] and quantity of dietary fat [27] in relation to obesity. Of these studies, no significant association was found between obesity and types of cooking fat and oil [23, 47]. These studies are limited in their capacity to reveal the relative proportions of dietary fatty acids that are consumed, therefore, they may not be able to inform nutrition policy formulation. Moreover, dietary energy intake was not adjusted in these studies.

There was an inverse association between general obesity and WC with ∑ω-3/∑ω-6 ratio in the present study. Contrary, Lund et al. [19] in the Danish cohort study, found non-significant associations between ∑ω-3/∑ω-6 ratio and WC and BMI. The disparity between our findings and Lund et al. study might be explained by the differences in study design, sample size and characteristics of study participants. Results from cross-sectional studies in Alabama [20] and Algeria [18] showed inverse associations between ∑ω-6/∑ω-3 ratios and obesity. In contrast, this argues against the present study results and also the findings from a review [48], in which the author clearly reported that a higher ratio of ∑ω-6/∑ω-3 increases the risk of obesity.

In the present study, there was a positive association between TF and abdominal obesity. In contrast to our observation, two previous cross-sectional studies from Ghana reported inverse associations between the percentage of energy obtained from total fat and overweight/obesity [16, 49]. However, Mogre et al. showed a weak inverse association between the absolute quantity of dietary fat intake and both WC and BMI among University students [27]. The reasons for the disparity between the present findings and these earlier studies are not understood, however, differences in the study settings may have partly introduced the variations in the results. Moreover, a setback in those earlier studies was that the effect of total energy intake was not adjusted in the analysis.

In the present study, the findings revealed a positive relationship between TI and LI and a chance for both general and abdominal obesity as well as a positive association between AI and TF and abdominal obesity. It is not entirely understood what accounted for the positive associations observed between obesity and the above DFQ indices, however, it might have resulted from the cumulative contributions of palmitic, myristic and stearic acids and possibly the activities of linoleic acid (LA). This is because higher values of AI, TI and LI favor greater intakes of palmitic, myristic and stearic acids [30, 32], which are linked to increased risk of obesity [14].

Higher intake of PSR was found to associate inversely with general and abdominal obesity although this observation did not attain statistical significance. The present findings compare partly with the results of two prospective studies [12, 18], in which Phillips and colleagues [12] showed that, low dietary PUFA:SFA ratio at baseline significantly associated with increased risk of both general and abdominal obesity whereas, low dietary PUFA:SFA ratio at follow-up only significantly related with higher increased risk of general obesity but showed non-significant positive association with abdominal obesity. Another prospective study from Africa [18] revealed lower intake of unsaturated fatty acids to SFA ratio in overweight/obese T2DM subjects and overweight/obese subjects without T2DM compared with T2DM of normal weight.

Although the mechanisms involved in the association of fatty acids and obesity are not clear, there is evidence that long-chain omega fatty acids have the capacity to suppress appetite while prolonging satiety [50]. Their ingestion also influences gene expressions in various organs that suppress fat deposition, cause an increase in both β-oxidation and energy expenditure [51, 52]. Similarly, the medium chain fatty acids are said to cause increased fat oxidation and energy expenditure [53, 54]. On the other hand, higher intake of long-chain SFAs such as myristic, palmitic and stearic acids tend to favor higher values of LI, AI and TI [31, 32]. They have the ability to influence gene expressions in various organs more especially fat mass and obesity-associated gene and signal transducer and activator of transcription 3 gene polymorphisms [12, 13, 55], thereby, increasing the risk of obesity. Similarly, high ω-6 fatty acids intake may promote the development of obesity. For instance, evidence from animal studies suggested that when linoleic acid is converted to arachidonic acid, it has the capacity to engineer body weight gain and adipogenesis partly through the prostacyclin pathway [56].

### Strengths and limitations

The present study has important strengths. First, we adjusted for the effects of total energy intake and other important confounding variables in order to estimate independent associations between fat indices and obesity. Second, as far as we know, this was the first study to investigate DFQ in such a broader form and its relationship with general and abdominal obesity in Ghana; this may serve as precedence for other researchers. Despite these strengths, the study has some limitations. First, the sample size was small. Second, due to the cross-sectional design of the study, our findings do not proclaim a causal relationship between fat indices and obesity/overweight. Therefore, the present results ought to be interpreted with great caution. Third, dietary intake assessment was based on recalls thus, a chance for misreporting of food items cannot be ruled out completely. Moreover, despite the fact that we adjusted for the effects of dietary energy and other important potential confounders, residual confounding cannot be ruled out. Moreover, the study duration was short. Hence, we cannot discount seasonal effects on food intake.

## Conclusion

The present study revealed an inverse association between ∑ω-3 / ∑ω-6 ratio, and general obesity and WC. Furthermore, abdominal obesity showed a positive association with AI and TF. Additionally, both general and abdominal obesity had positive relationships with TI and LI. Therefore, dietary advice in favor of an increased higher ratio of ∑ω-3 / ∑ω-6, and a lower intake of AI, LI, and TF may be useful for obesity prevention. The effect of fat quality on obesity should be further studied using an experimental design.

## Data Availability

The datasets used to support the findings of this study are available from the corresponding author upon request.

## Abbreviations

DFQ: Dietary fat quality
PSR: Polyunsaturated to saturated fatty acids ratio
h/H: Hypo and hypercholesterolemic ratio
AI: Atherogenicity index
TI: Thrombogenic index
LI: Lipophilic index
TF: Percentage of energy from total fat
T2DM: Type 2 diabetes mellitus
MET-minutes/weeks: Metabolic equivalent minutes per week
12:0: Lauric acid
14:0: Myristic acid
16:0: Palmitic acid
18:0: Stearic acid
18:1 ω-9: Oleic acid
18:2 ω-6: Linoleic acid
20:4 ω-6: Arachidonic acid
18:3 ω-3: Alpha-linolenic acid
20:5 ω-3: Eicosapentaenoic acid
22:5 ω-3: Docosapentaenoic acid
22:6 ω-3: Docosahexaenoic acid
MUFA: Monounsaturated fatty acids
PUFA: Polyunsaturated fatty acids
SFAs: Saturated fatty acids
∑ω-6 PUFA: Sum of omega-6 polyunsaturated fatty acids
∑ω-3 PUFA: Sum of omega-3 polyunsaturated fatty acids
kcal/d: kilocalories per day
Qs: Quintiles
BMI: Body Mass Index
WC: Waist circumference
WHtR: Waist to Height Ratio
OR: Odds ratio
P: Statistical significance level for trend test
SDs: Means ± standard deviations
CIs: Confidence intervals
LA: Linoleic acid
